# Assessment of the relationship between gut microbiota and bone mineral density: a two-sample Mendelian randomization study

**DOI:** 10.3389/fmicb.2024.1298838

**Published:** 2024-05-22

**Authors:** Yuan Xue, Xuan Wang, Honglin Liu, Junfeng Kang, Xiaohong Liang, Aina Yao, Zhifang Dou

**Affiliations:** ^1^Graduate School, College of Basic Medical Sciences, Shanxi University of Traditional Chinese Medicine, Taiyuan, China; ^2^Department of Traditional Chinese Medicine, Second Affiliated Hospital of Xi’an Jiaotong University, Xi’an, China; ^3^Dean’s Office, Shanxi Vocational College of Health, Taiyuan, China; ^4^Department of Orthopedics, Affiliated Hospital of Shanxi University of Traditional Chinese Medicine, Taiyuan, China; ^5^Department of Brain Disease, Shanxi Acupuncture and Moxibustion Hospital, Taiyuan, China

**Keywords:** gut microbiota, bone mineral density, mendelian randomization, causal relationship, osteoporosis

## Abstract

**Background:**

Emerging evidence from observational studies and clinical trials suggests a connection between the gut microbiota and variations in bone mineral density (BMD). Nonetheless, the specific association between gut microbiota and BMD alterations at different skeletal sites has not been comprehensively explored. To address this, we employed Genome-Wide Association Study (GWAS) summary statistics from a publicly accessible database, conducting a two-sample Mendelian Randomization analysis to elucidate the potential causal relationship between gut microbiota composition and BMD.

**Methods:**

This study utilized two distinct thresholds for screening instrumental variables (IVs), followed by an extensive series of quality control procedures to identify IVs that were significantly related to exposure. Gut microbiota were classified into two sets based on hierarchical levels: phylum, class, order, family, and genus. Bone mineral density (BMD) data were systematically collected from four skeletal sites: femoral neck, lumbar spine, forearm, and heel. For Mendelian Randomization (MR) analysis, robust methods including Inverse-Variance Weighting (IVW) and the Wald Ratio Test were employed. Additional analytical tests such as the Outlier Test, Heterogeneity Test, ‘Leave-One-Out’ Test, and Pleiotropy Test were conducted to assess the impact of horizontal pleiotropy, heterogeneities, and the genetic variation stability of gut microbiota on BMD causal associations. The MR Steiger Directionality Test was applied to exclude studies with potential directional biases.

**Results:**

In this two-sample Mendelian randomization analysis, we utilized five sets of exposure GWAS (Genome-Wide Association Studies) summary statistics and four sets of outcome GWAS summary statistics. The initial analysis, applying a threshold of *p* < 5 × 10^−6^, identified 48 significant causal relationships between genetic liability in the gut microbiome and bone mineral density (BMD). A subsequent analysis with a more stringent threshold of *p* < 5 × 10^−8^ uncovered 14 additional causal relationships. Upon applying the Bonferroni correction, 9 results from the first analysis and 10 from the second remained statistically significant.

**Conclusion:**

Our MR analysis revealed a causal relationship between gut microbiota and bone mineral density at all sites, which could lead to discoveries in future mechanistic and clinical studies of microbiota-associated osteoporosis.

## Introduction

Osteoporosis, a systemic skeletal metabolic disease, predominantly impacts the elderly and is characterized by reduced bone mineral density (BMD) and deteriorated bone tissue microstructure (Notelovitz, 1993). Osteoporosis also is a bone disease that increases the risk of fractures due to reduced bone density and quality (NIH Consensus Development Panel on Osteoporosis Prevention, Diagnosis, and Therapy, 2001). Clinically, BMD serves as an indirect marker for osteoporosis and associated fracture risk. Osteoporosis and its associated fragility fractures have a significant impact on mortality and morbidity in individuals, healthcare systems, and entire communities (Clynes et al., 2020). A 2010 epidemiological study (Wright et al., 2014) in the United States revealed that approximately 5 million elderly Americans suffered from low bone mass or osteoporosis, particularly at the femoral neck and lumbar spine, with over 80% exhibiting low bone mass. The prevalence of osteoporosis-related fractures imposes a considerable burden on public health, healthcare systems, and the economy (Darbà et al., 2015; Fischer et al., 2017; Aziziyeh et al., 2019). According to the findings of an epidemiologic survey of 29 countries (including 27 in the European Union, Switzerland, and the United Kingdom), the economic burden of fragility fractures was € 5.7 billion in 2019, resulting in more than 200,000 deaths. The number of osteoporotic fractures in these countries is expected to rise by 25% annually until 2034 (Willers et al., 2022).

The gut microbiota, a diverse assembly of microorganisms in the gastrointestinal tract, encompasses bacteria, yeast, and various other microorganisms (Rinninella et al., 2019). Numerous studies have established a connection between gut microbiota composition and bone mineral density (BMD) development. These bacterial species within the gut microbiota are taxonomically categorized across genus, family, order, and phyla (Rinninella et al., 2019). Research indicates that an imbalance in gut microbiota, or dysbiosis, is linked to a range of diseases, encompassing both intestinal disorders such as inflammatory bowel disease and celiac disease, as well as extraintestinal conditions including metabolic syndrome and obesity (Carding et al., 2015; Rinninella et al., 2019). The influence of gut microbiota extends to disease susceptibility and the effectiveness of medications, suggesting its potential role in addressing unresolved biological and infectious disease challenges (Nicholson et al., 2012).

In the burgeoning field of microbiome research, the influence of gut microbiota on bone health has garnered considerable attention. Research indicates that individuals with osteoporosis and bone loss exhibit distinct changes in the composition and diversity of gut bacteria compared with healthy controls (Wang et al., 2017; Li et al., 2019; He et al., 2020; Rettedal et al., 2021). Studies, involving germ-free mice, which display increased bone mineral density (BMD) relative to conventional mice, and the observed normalization of bone mass following gut microbiota colonization, provide evidence supporting the regulatory role of gut microbiota in BMD (Sjogren et al., 2012). These findings collectively suggest the existence of a gut microbiota–bone axis, further underlining the connection between gut microbiota composition and BMD development (He and Chen, 2022).

As an experimental design for detecting associations between genetic variants and traits in population samples (Visscher et al., 2017), genome-wide association studies (GWAS) are a powerful method for identifying genes associated with common human diseases (The Wellcome Trust Case Control Consortium, 2007). GWAS have become the most successful method for identifying variants and genes that have a significant impact on BMD in recent years (Martinez-Gil et al., 2023). In the past, more than 50 large-scale GWAS or GWAS meta-analyses published 500 genetic loci associated with various bone parameters (e.g., bone mineral density) (Martinez-Gil et al., 2023). The clinical application of GWAS data allows for research on the susceptibility, prevention, and treatment of low bone mass and osteoporosis.

It is well understood that the results of randomized controlled trials are more capable of establishing causal links. Unfortunately, the majority of current relevant studies are based on RNA sequencing methods to examine the composition and changes in the gut microbiota in feces of patients with bone loss (Li et al., 2019; Ozaki et al., 2021), which does not provide a good indication of a causal link (He and Chen, 2022). This Mendelian randomization (MR) study was carried out to investigate the causal relationships between gut microbiota and body site bone mineral density. MR is a method for improving causal inference by using genetic variations as an indicator of exposure while avoiding the limitations of traditional study designs (Smith and Ebrahim, 2003; Smith and Ebrahim, 2004; Timpson et al., 2012). MR is a widely used method for exploring potential causal relationships between environmental exposure and disease, and it is designed to improve causal inferences by analyzing inherent properties of common genetic variations for a modifiable environmental exposure of interest (Smith and Ebrahim, 2003; Davey Smith and Hemani, 2014; Sekula et al., 2016).

In this study, bone mineral density (BMD) data were analyzed from three distinct skeletal sites, namely, the femoral neck, forearm, and lumbar spine, which were sourced from the Genetic Factors for Osteoporosis Consortium (GEFOS) website, complemented by heel BMD data from 426,824 participants in the United Kingdom Biobank (UKB). This comprehensive investigation aimed to elucidate the causal relationship between gut microbiota and BMD alterations at multiple skeletal sites in the human body. The findings have the potential to clarify the interaction between various gut microbiota and BMD, offering insights for developing new therapeutic strategies and treatment methods for osteoporosis that leverage gut microbiota.

## Methods

### Exposure data

As instrumental variables in the exposure data, we used SNPs from GWAS data in the International Consortium MiBioGen.^1^ The MiBioGen consortium currently has the most comprehensive study of host-genetics-versus-microbiome associations, with the largest sample size and geographic scope of the study (Wang et al., 2018). In the study, the 16S ribosomal RNA (rRNA) gene sequencing profiles and genotyping data of 18,340 participants came from the United States, Canada, Israel, South Korea, Germany, Denmark, the Netherlands, Belgium, Sweden, Finland, and the United Kingdom (Kurilshikov et al., 2021). The flora was classified into 131 genera, 35 families, 20 orders, 16 classes, and 9 phyla.

### Outcome data

The GWAS summary statistics for BMDs (unit, g/cm2) were obtained from the website of the Genetic Factors for Osteoporosis Consortium.^2^ The data are based on the use of the Dual Energy X-ray Bone Densitometer (DXA) to measure femoral neck bone mineral density (FN-BMD, *n* = 32,735), forearm bone mineral density (FA-BMD, *n* = 8,143), and lumbar spine bone mineral density (LS-BMD, *n* = 28,498) for men of older than 50 years and postmenopausal women. To date, this is the largest GWAS based on DXA-measured BMD (Zheng et al., 2015).

In addition, from the GEFOS website, we downloaded and used heel bone mineral density (HE-BMD) data from 426,824 UKB cohort participants (Morris et al., 2019). The UKB cohort included approximately 50,000 people aged 40 to 69 years. The quantitative ultrasound speed of sound (SOS) and broadband ultrasound attenuation were used to calculate HE-BMD. The IEU GWAS database^3^ contains all of the above GWAS summary statistics for BMD, and all participants were of European origin.

### Selection of instrumental variables

**Figure 1:**
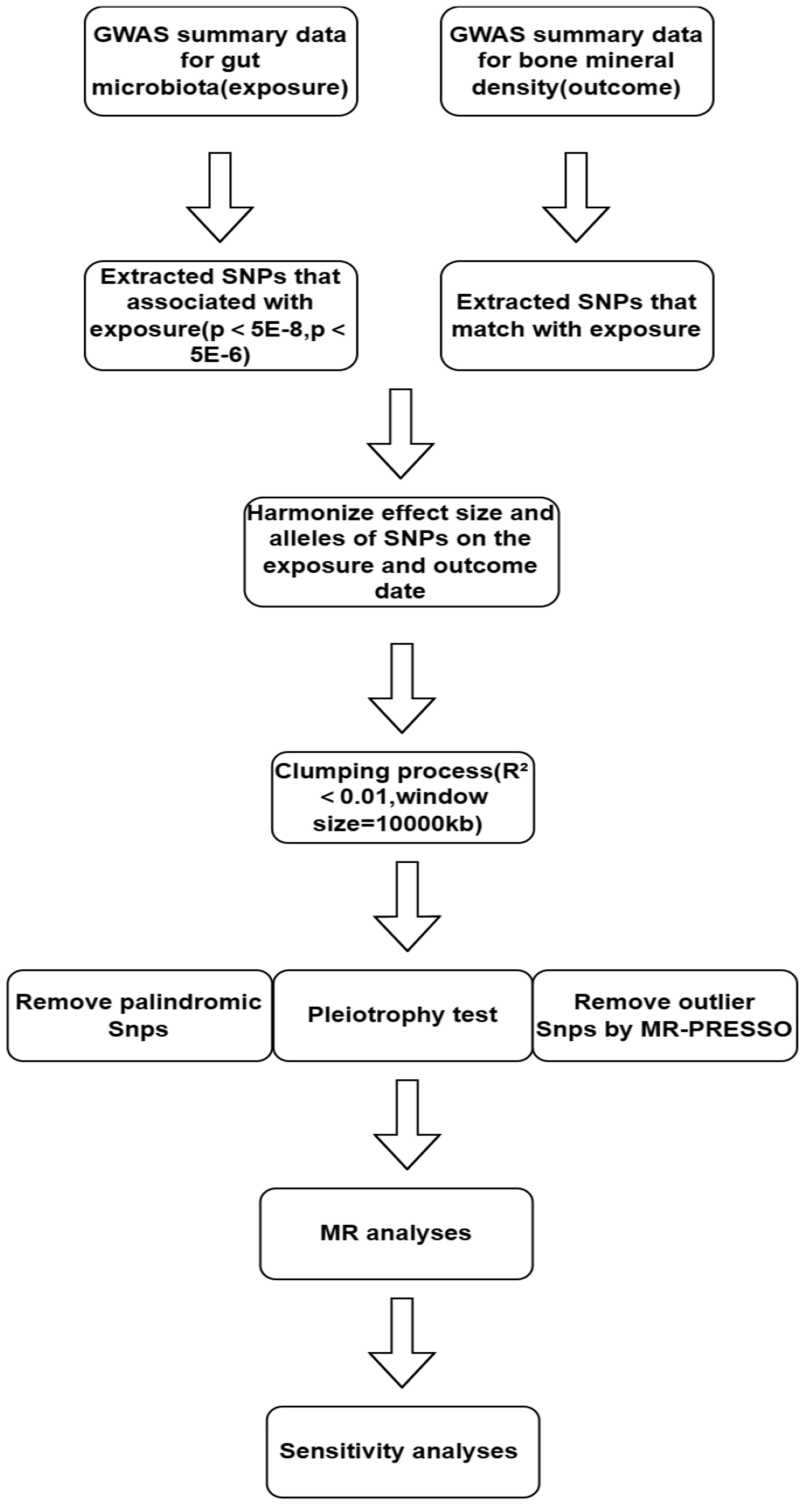
Flow chart.

Figure 1 illustrates the workflow of the study. We independently analyzed the five taxonomic levels of gut microbiota in the exposure group (genera, families, orders, classes, and phyla) in relation to BMD outcomes. To ensure the validity of the study, a rigorous quality control process was implemented prior to commencing Mendelian randomization (MR) analysis. Single Nucleotide Polymorphisms (SNPs) significantly associated with gut microbiota were initially selected as instrumental variables (IVs) using a genome-wide significance threshold of 5 × 10^−8^. However, this stringent threshold yielded only a limited number of gut microbiota.

To investigate the causal relationship between gut flora and BMD in greater depth, we used (5 × 10^−6^) as the second threshold and selected SNPs smaller than the genome-wide statistical significance threshold (5 × 10^−6^) as the second set of IVs. We removed LD (clumping process:*R*^2^ < 0.01 and clumping distance = 10,000 kb) because strong linkage disequilibrium (LD) may cause bias in the results. At the same time, we removed palindromic SNPs (e.g., A/T or G/C alleles) to ensure that the effects of SNPs on exposure and results correspond to the same allele and avoid distortion of strand orientation or allele coding in MR.

We utilized the MR-PRESSO framework to conduct an outlier test, which enabled the identification of aberrant SNPs (outliers significantly divergent from other SNPs) in the exposure. Outliers identified through this test were subsequently excluded. Additionally, we executed the MR pleiotropy test to assess the presence of horizontal pleiotropy in individual instrumental variables (IVs). If the MR-Egger intercept does not significantly differ from zero (*p*-value >0.05), it indicates the absence of horizontal pleiotropy. In cases where horizontal pleiotropy is detected, prior to conducting further MR analysis, the PhenoScanner database^4^ (Keller-Baruch et al., 2020) is utilized to identify and exclude SNPs associated with potential confounders (such as diet, frailty variables, physical activity levels, medications, weight, BMI, gender, and bone density measurements) until the pleiotropy is no longer statistically significant.

### MR analysis

The current MR analysis focused on the causal relationship of microbiome features with BMD at four skeletal sites in humans. The Wald ratio test was used to estimate the causal relationship of microbiome features with BMD (when features contained only one IV) (Burgess et al., 2017). When features contain multiple IVs, five established MR methods are available, namely, the inverse-variance weighted (IVW) test (Burgess et al., 2013), the weighted mode (Hartwig et al., 2017), the MR-Egger regression (Bowden et al., 2015), the weighted median estimator (WME) (Bowden et al., 2016), and the MR-PRESSO (Verbanck et al., 2018). However, the IVW method is reported to outperform the other methods under certain conditions and is currently the most accurate method for estimating causality (Bowden et al., 2016; Holmes et al., 2017), so it was primarily used in our study of multiple IVs.

To ensure the robustness of our findings, we conducted a leave-one-out analysis to determine if individual SNPs significantly influenced the results (Burgess and Thompson, 2017). SNPs demonstrating a substantial impact were excluded prior to conducting Mendelian randomization (MR) analysis. An SNP was deemed to significantly alter the results if its central value in the leave-one-out plot exhibited a trend contrary to the overall findings. Furthermore, to identify any weak instrument variables, we calculated the F-statistic for each instrumental variable (IV) related to the exposure using the formula: F = β^2^_exposure_/SE^2^_exposure_.

If the F statistic is significantly greater than 10, a weak IV bias is highly unlikely (Staiger and Stock, 1997). Individual IVs with *F* values of less than 10 are regarded as weak IVs. On this basis, we also performed the MR Steiger directionality test to observe if the directionality of the MR results matches our assumption of the correct direction of the results; if the directionality is incorrect, the group of exposures and outcomes is excluded from our MR analysis. In addition, we used the two-sample MR package to run the heterogeneity test to look at the differences between the individual IVs. There was no heterogeneity if Q statistics were significant at *p*-value >0.05.

Furthermore, we used Bonferroni correction to establish a multiple testing significance threshold at each level of the gut microbiota (phylum, class, order, family, and genus). The p-value was calculated as p 0.05/n, where n is the effective number of independent bacterial taxa at each taxonomic level. The significance thresholds for the different taxa levels were set to the following values when the threshold was less than 5 × 10^−8^: class *p* = 0.05 (0.05/1), order *p* = 0.025 (0.05/2)，family *p* = 1.6 × 10–2(0.05/3), and genus *p* = 6.3 × 10–3(0.05/8). The significance thresholds for the different taxa levels were set to the following values when the threshold was less than 5 × 10^−6^: phylum *p* = 0.05 (0.05/1), class *p* = 0.025 (0.05/2), order *p* = 1.3 × 10^−2^(0.05/4), family *p* = 5.5 × 10^−3^(0.05/9), and genus *p* = 1.8 × 10^−3^(0.05/28). Although the Bonferroni correction method is debatable (Rothman, 1990; Armstrong, 2014), it reduces the likelihood of type I error in numerous statistical tests (Staiger and Stock, 1997) and can produce more reliable results.

## Results

### SNP selection

The determination of the number of Single Nucleotide Polymorphisms (SNPs) utilized in the analysis was based on a predefined threshold value (*p* < 5 × 10^-8), which was coupled with multiple screenings and stringent quality control measures. Specifically, for the femoral neck-bone mineral density (FN-BMD) group, one SNP was selected; for the Forearm BMD (FA-BMD) group, two SNPs were selected; for the Lumbar Spine BMD (LS-BMD) group, four SNPs were selected; and for the Heel BMD (HE-BMD) group, seven SNPs were selected. In a similar vein, setting a threshold value of *p* < 5 × 10^-6, along with multiple screenings and rigorous quality control, led to the selection of 50 SNPs for the FN-BMD group, 81 SNPs for the FA-BMD group, 72 SNPs for the LS-BMD group, and 52 SNPs for the HE-BMD group.

In the Mendelian randomization (MR) analysis, all instrumental variables (IVs) demonstrated an F statistic exceeding 10, as shown in Supplementary Table S1. This result suggests the absence of weak instrumental variable bias in the study. Outlier IVs were identified and removed using the MR-PRESSO test. Notably, in the MR analysis focusing on the genus *Turicibacter* and its association with HE-BMD, three outlier SNPs were detected (rs11054680, rs149744580, and rs55756211), with an IV significance threshold set at *p* < 5 × 10^-6.

### The causal relationship between gut microbiota and BMD of human four skeletal sites

#### Femoral neck bone mineral density

**Figure 2:**
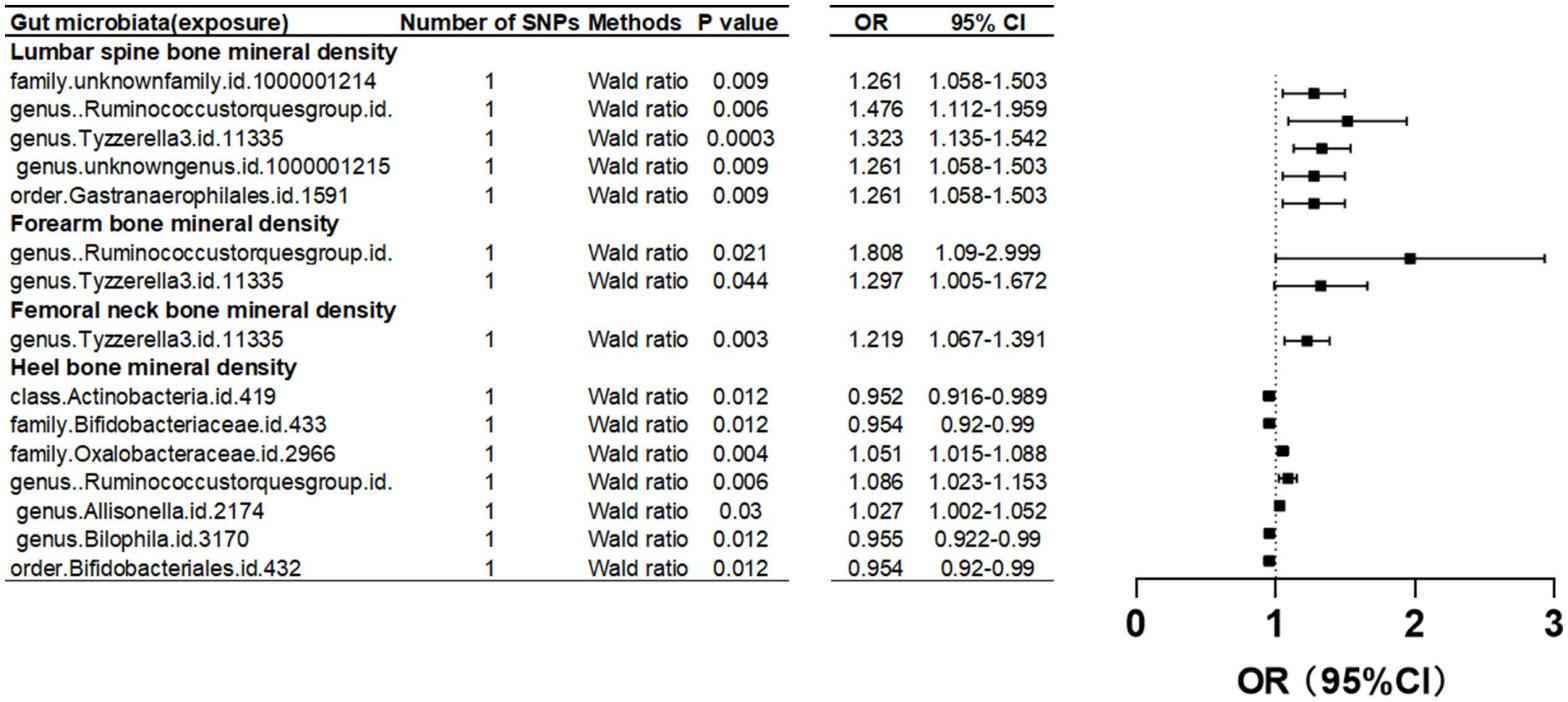
Mendelian randomization results of causal effects between gut microbiome and BMD (*p*<5 × 10^−8^).

Upon setting the instrumental variable (IV) threshold at *p* < 5 × 10^-8, our analysis identified several gut microbiota causally associated with femoral neck bone mineral density (FN-BMD). At the genus level, Tyzzerella3 was implicated (Figure 2; Table 1).

**Table 1:** Mendelian randomization (MR) results of causal effects between gut microbiome and BMD (*p* < 5 × 10^−8^).

Gut microbiota (exposure)	Bone density reduction sites(outcomes)	Method	Number of SNPS	*β*	SE	*P*-value	OR	95%CI	Correct causal direction	Steiger *p*-value
Lumbar spine bone mineral density										
Family.unknownfamily.id.1000001214	Lumbar spine	Wald ratio	1	2.32E-01	8.95E-02	0.009	1.261	1.058–1.503	TRUE	1.89E-05
Genus.Ruminococcustorquesgroup.id.14377	Lumbar spine	Wald ratio	1	3.90E-01	1.44E-01	0.006	1.476	1.112–1.959	TRUE	8.82E-04
Genus.Tyzzerella3.id.11335	Lumbar spine	Wald ratio	1	2.80E-01	7.81E-02	0.0003	1.323	1.135–1.542	TRUE	6.55E-06
Order.Gastranaerophilales.id.1591	Lumbar spine	Wald ratio	1	2.32E-01	8.95E-02	0.009	1.261	1.058–1.503	TRUE	1.89E-05
Forearm bone mineral density										
Genus.Ruminococcustorquesgroup.id.14377	Forearm	Wald ratio	1	5.92E-01	2.58E-01	0.021	1.808	1.09–2.999	TRUE	9.45E-02
Genus.Tyzzerella3.id.11335	Forearm	Wald ratio	1	2.60E-01	1.30E-01	0.044	1.297	1.005–1.672	TRUE	1.45E-04
Femoral neck bone mineral density										
Genus.Tyzzerella3.id.11335	Femoral neck	Wald ratio	1	1.98E-01	6.76E-02	0.003	1.219	1.067–1.391	TRUE	1.67E-06
Heel bone mineral density										
Class.Actinobacteria.id.419	Heel	Wald ratio	1	−4.88E-02	1.95E-02	0.012	0.952	0.916–0.989	TRUE	9.45E−18
Family.Bifidobacteriaceae.id.433	Heel	Wald ratio	1	-4.65E-02	1.86E-02	0.012	0.954	0.92–0.99	TRUE	7.06E−18
Family.Oxalobacteraceae.id.2966	Heel	Wald ratio	1	5.02E-02	1.76E-02	0.004	1.051	1.015–1.088	TRUE	3.22E-07
Genus.Ruminococcustorquesgroup.id.14377	Heel	Wald ratio	1	8.32E-02	3.06E-02	0.006	1.086	1.023–1.153	TRUE	7.60E-07
Genus.*Allisonella*.id.2174	Heel	Wald ratio	1	2.71E-02	1.25E-02	0.03	1.027	1.002–1.052	TRUE	4.47E-08
Genus.Bilophila.id.3170	Heel	Wald ratio	1	-4.54E-02	1.82E-02	0.012	0.955	0.922–0.99	TRUE	1.66E−18
Order.Bifidobacteriales.id.432	Heel	Wald ratio	1	-4.65E-02	1.86E-02	0.012	0.954	0.92–0.99	TRUE	7.06E−18

**Figure 3:**
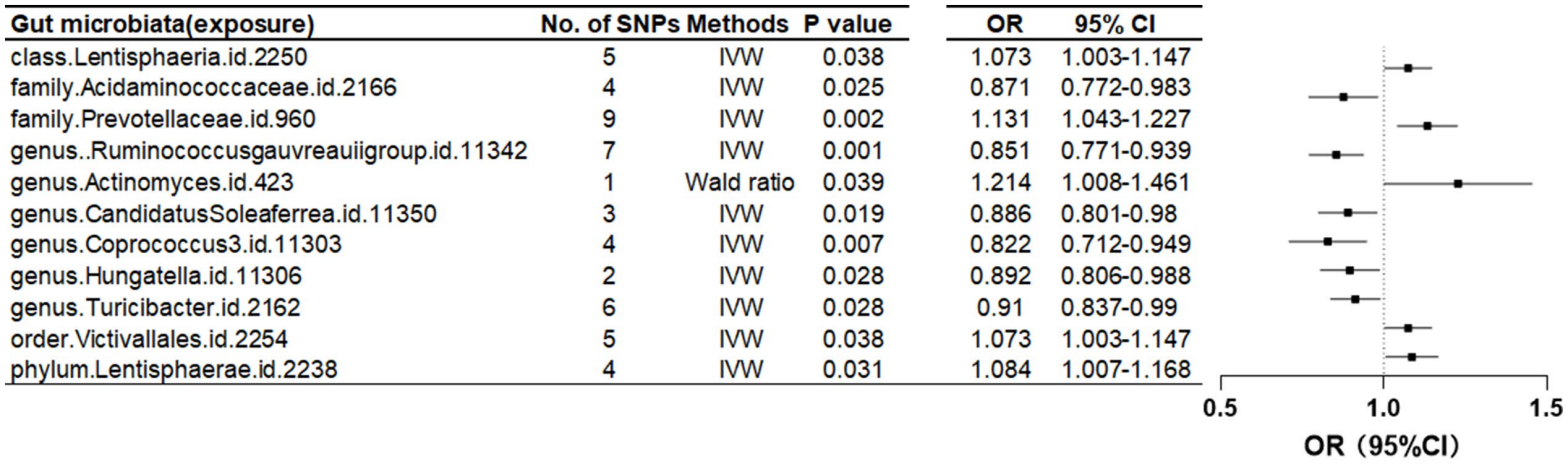
Mendelian randomization results of causal effects between gut microbiome and femoral neck BMD (*p* < 5 × 10^−6^).

Further investigation revealed a class Lentisphaeria and two families, Prevotellaceae and Acidaminococcaceae, as having causal associations with FN-BMD. Additionally, at the genus level, the *Ruminococcus gauvreauii* group, Actinomyces, *Candidatus* Soleaferrea, *Coprococcus*, *Hungatella*, and *Turicibacter* were identified as contributing factors. Notably, causal relationships with FN-BMD were also observed at the order and phylum levels, specifically with Victivallales (order) and Lentisphaerae (phylum) as presented in Table 2 and Figure 3.

**Table 2:** Mendelian randomization (MR) results of causal effects between gut microbiome and femoral neck BMD (*p* < 5 × 10^−6^).

Gut microbiota (exposure)	Bone density reduction sites(outcomes)	Method	Number of SNPS	*β*	SE	*P*-value	OR	95%CI	Correct causal direction	Steiger *p*-value	Q	Q_df	Q_pval
Class.Lentisphaeria.id.2250	Femoral neck	IVW	5	7.08E-02	3.43E-02	0.038	1.073	1.003–1.147	TRUE	2.04E-21	1.926	4	0.749
Family.Acidaminococcaceae.id.2166	Femoral neck	IVW	4	−1.37E-01	6.17E-02	0.025	0.871	0.772–0.983	TRUE	3.73E-14	2.609	3	0.455
Family.Prevotellaceae.id.960	Femoral neck	IVW	9	1.24E-01	4.14E-02	0.002	1.131	1.043–1.227	TRUE	6.06E-38	3.821	8	0.872
Genus.Ruminococcusgauvreauiigroup.id.11342	Femoral neck	IVW	7	−1.61E-01	5.01E-02	0.001	0.851	0.771–0.939	TRUE	1.20E-20	6.493	6	0.37
Genus.Actinomyces.id.423	Femoral neck	Wald ratio	1	1.94E-01	9.45E-02	0.039	1.214	1.008–1.461	TRUE	2.56E-04	NA	NA	NA
Genus.CandidatusSoleaferrea.id.11350	Femoral neck	IVW	3	−1.20E-01	5.16E-02	0.019	0.886	0.801–0.98	TRUE	5.56E-12	0.554	2	0.757
Genus.Coprococcus3.id.11303	Femoral neck	IVW	4	−1.95E-01	7.34E-02	0.007	0.822	0.712–0.949	TRUE	4.95E-12	0.9	3	0.825
Genus.Hungatella.id.11306	Femoral neck	IVW	2	−1.13E-01	5.18E-02	0.028	0.892	0.806–0.988	TRUE	9.26E-09	0.419	1	0.517
Genus.Turicibacter.id.2162	Femoral neck	IVW	6	−9.36E-02	4.27E-02	0.028	0.91	0.837–0.99	TRUE	7.52E-23	4.988	5	0.417
Order.Victivallales.id.2254	Femoral neck	IVW	5	7.08E-02	3.43E-02	0.038	1.073	1.003–1.147	TRUE	2.04E-21	1.926	4	0.749
Phylum.Lentisphaerae.id.2238	Femoral neck	IVW	4	8.12E-02	3.78E-02	0.031	1.084	1.007–1.168	TRUE	1.18E−17	1.479	3	0.686

### Forearm bone mineral density

In this study, we found that the gut microbiota genus *Ruminococcus torques* group and genus *Tyzzerella* are causally associated with FA-BMD when the instrumental variable (IV) threshold was set at *p* < 5 × 10^-8 (Figure 2; Table 1).

**Figure 4:**
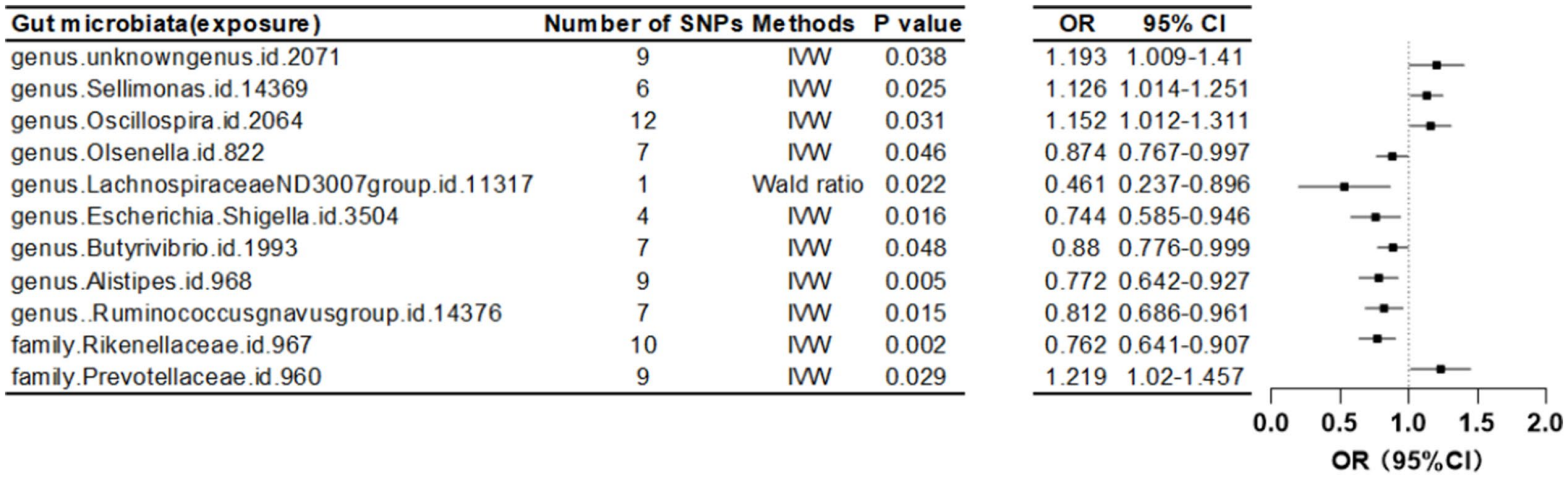
Mendelian randomization results of causal effects between gut microbiome and forearm BMD (*p* < 5 × 10^−6^).

Furthermore, at a threshold of *p* < 5 × 10^-6, nine gut microbiota were identified as causally associated with FA-BMD. Seven of these belonged to the genus level, including an unknown genus id 2071, *Sellimonas*, *Oscillospira*, *Olsenella*, *Lachnospiraceae* ND3007 group, *Escherichia Shigella*, *Butyrivibrio*, *Alistipes*, and *Ruminococcus gnavus* group. The remaining two, belonging to the family level, were Rikenellaceae and Prevotellaceae (Table 3; Figure 4).

**Table 3:** Mendelian randomization (MR) results of causal effects between gut microbiome and forearm BMD (*p* < 5 × 10^−6^).

Gut microbiota (exposure)	Bone density reduction sites(outcomes)	Method	Number of SNPS	*β*	SE	*P*-value	OR	95%CI	Correct causal direction	Steiger *p*-value	Q	Q_df	Q_pval
Genus.unknowngenus.id.2071	Forearm	IVW	9	1.77E-01	8.52E-02	0.038	1.193	1.009–1.41	TRUE	4.78E-16	3.888	8	0.867
Genus.*Sellimonas*.id.14369	Forearm	IVW	6	1.19E-01	5.33E-02	0.025	1.126	1.014–1.251	TRUE	3.36E-19	5.019	5	0.413
Genus.*Oscillospira*.id.2064	Forearm	IVW	12	1.42E-01	6.59E-02	0.031	1.152	1.012–1.311	TRUE	7.64E-26	6.675	11	0.824
Genus.*Olsenella*.id.822	Forearm	IVW	7	−1.34E-01	6.71E-02	0.046	0.874	0.767–0.997	TRUE	1.78E-19	8.635	6	0.195
Genus.*Lachnospiraceae*ND3007group.id.11317	Forearm	Wald ratio	1	−7.74E-01	3.39E-01	0.022	0.461	0.237–0.896	TRUE	1.69E-01	NA	NA	NA
Genus.*Escherichia*.*Shigella*.id.3504	Forearm	IVW	4	−2.95E-01	1.23E-01	0.016	0.744	0.585–0.946	TRUE	8.58E-07	0.495	3	0.919
Genus.*Butyrivibrio*.id.1993	Forearm	IVW	7	−1.27E-01	6.42E-02	0.048	0.88	0.776–0.999	TRUE	1.08E−20	9.564	6	0.144
Genus.*Alistipes*.id.968	Forearm	IVW	9	−2.58E-01	9.36E-02	0.005	0.772	0.642–0.927	TRUE	8.34E-07	3.414	8	0.905
Genus.Ruminococcusgnavusgroup.id.14376	Forearm	IVW	7	−2.08E-01	8.61E-02	0.015	0.812	0.686–0.961	TRUE	2.78E−14	9.789	6	0.133
Family.Rikenellaceae.id.967	Forearm	IVW	10	−2.71E-01	8.84E-02	0.002	0.762	0.641–0.907	TRUE	7.84E−13	5.79	9	0.76
Family.*Prevotellaceae*.id.960	Forearm	IVW	9	1.98E-01	9.09E-02	0.029	1.219	1.02–1.457	TRUE	4.78E−16	8.878	8	0.352

### Lumbar spine bone mineral density

In the study, at an instrumental variable (IV) threshold of *p* < 5 × 10^-8, the gut microbiota found to be causally associated with lumbar spine bone mineral density (LS-BMD) including an unidentified family (id 1,000,001,214), genus *Ruminococcus torques* group, genus *Tyzzerella3*, and order Gastranaerophilales (Figure 2; Table 1).

**Figure 5:**
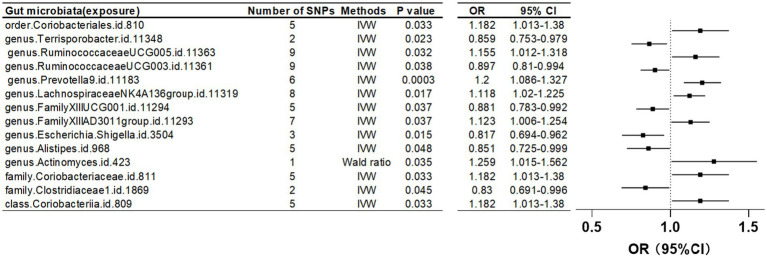
Mendelian randomization results of causal effects between gut microbiome and lumbar spine BMD (*p* < 5 × 10^−6^).

Furthermore, when the IV threshold was set at *p* < 5 × 10^-6, 19 gut microbiota were identified as having a causal association with LS-BMD. These included the class Coriobacteriia, two families (Clostridiaceae1 and Coriobacteriaceae), and 10 genera: *Actinomyces*, *Alistipes*, *Escherichia Shigella*, Family XIII AD3011 group, Family XIIIUCG-001, Lachnospiraceae NK4A136 group, *Prevotella9*, Ruminococcaceae UCG-003, Ruminococcaceae UCG-005, and *Terrisporobacter*. Additionally, one order, Coriobacteriales, was also found to have a causal relationship (Table 4; Figure 5).

**Table 4:** Mendelian randomization (MR) results of causal effects between gut microbiome and lumbar spine BMD (*P* < 5 × 10^−6^).

Gut microbiota (exposure)	Bone density reduction sites(outcomes)	Method	Number of SNPS	*β*	SE	*P*-value	OR	95%CI	Correct causal direction	Steiger *p*-value	Q	Q_df	Q_pval
Order.Coriobacteriales.id.810	Lumbar spine	IVW	5	1.68E-01	7.88E-02	0.033	1.182	1.013–1.38	TRUE	2.73E-14	2.005	4	0.7349
Genus.Terrisporobacter.id.11348	Lumbar spine	IVW	2	−1.52E-01	6.69E-02	0.023	0.859	0.753–0.979	TRUE	5.81E-09	NA	NA	NA
Genus.RuminococcaceaeUCG005.id.11363	Lumbar spine	IVW	9	1.44E-01	6.74E-02	0.032	1.155	1.012–1.318	TRUE	8.92E-35	14.26	8	0.0751
Genus.RuminococcaceaeUCG003.id.11361	Lumbar spine	IVW	9	−1.08E-01	5.22E-02	0.038	0.897	0.81–0.994	TRUE	3.47E-31	8.596	8	0.3775
Genus.*Prevotella*9.id.11183	Lumbar spine	IVW	6	1.83E-01	5.12E-02	0.0003	1.2	1.086–1.327	TRUE	2.51E−21	3.621	5	0.6051
Genus.LachnospiraceaeNK4A136group.id.11319	Lumbar spine	IVW	8	1.12E-01	4.68E-02	0.017	1.118	1.02–1.225	TRUE	3.67E-37	4.978	7	0.6627
Genus.FamilyXIIIUCG001.id.11294	Lumbar spine	IVW	5	−1.26E-01	6.05E-02	0.037	0.881	0.783–0.992	TRUE	6.52E−19	0.494	4	0.9741
Genus.FamilyXIIIAD3011group.id.11293	Lumbar spine	IVW	7	1.17E-01	5.62E-02	0.037	1.123	1.006–1.254	TRUE	8.18E-24	2.847	6	0.8278
Genus.*Escherichia*.*Shigella*.id.3504	Lumbar spine	IVW	3	−2.01E-01	8.33E-02	0.015	0.817	0.694–0.962	TRUE	1.52E-09	1.227	2	0.5414
Genus.*Alistipes*.id.968	Lumbar spine	IVW	5	−1.61E-01	8.18E-02	0.048	0.851	0.725–0.999	TRUE	1.89E-13	4.272	4	0.3704
Genus.*Actinomyces*.id.423	Lumbar spine	Wald ratio	1	2.31E-01	1.10E-01	0.035	1.259	1.015–1.562	TRUE	3.60E-04	NA	NA	NA
Family.Coriobacteriaceae.id.811	Lumbar spine	IVW	5	1.68E-01	7.88E-02	0.033	1.182	1.013–1.38	TRUE	2.73E-14	0.285	1	0.5934
Family.Clostridiaceae1.id.1869	Lumbar spine	IVW	2	−1.86E-01	9.31E-02	0.045	0.83	0.691–0.996	TRUE	5.11E-08	0.285	1	0.5934
Class.Coriobacteriia.id.809	Lumbar spine	IVW	5	1.68E-01	7.88E-02	0.033	1.182	1.013–1.38	TRUE	2.73E-14	2.005	4	0.7349

### Heel bone mineral density

At an instrumental variable (IV) threshold of p < 5 × 10^-8, our analysis identified that seven gut microbiota were causally associated with HE-BMD. These included the class Actinobacteria, families Bifidobacteriaceae and Oxalobacteraceae, the genera *Ruminococcus torques* group, *Allisonella*, and *Bilophila*, and the order Bifidobacteriales (Figure 2; Table 1).

**Figure 6:**
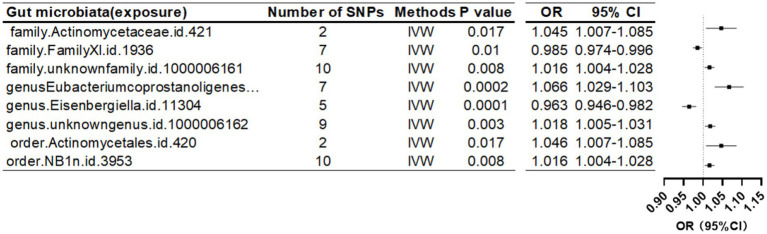
Mendelian randomization results of causal effects between gut microbiome and heel BMD (*p* < 5 × 10^−6^).

When the threshold was raised to p < 5 × 10^-6, eight additional gut microbiota were found to be causally linked to HE-BMD. These comprised three families: Actinomycetaceae, Family XI, and an unidentified family (id 1,000,006,161); three genera: *Eubacterium coprostanoligenes* group, *Eisenbergiella*, and an unknown genus (id 1,000,006,162); and two orders: Actinomycetales and NB1n (Table 5; Figure 6).

**Table 5:** Mendelian randomization (MR) results of causal effects between gut microbiome and heel BMD (*P* < 5 × 10^−6^).

Gut microbiota (exposure)	Bone density reduction sites(outcomes)	Method	Number of SNPS	*β*	SE	*P*-value	OR	95%CI	Correct causal direction	Steiger *p*-value	Q	Q_df	Q_pval
Family.Actinomycetaceae.id.421	Heel	IVW	2	4.49E-02	1.89E-02	0.017	1.045	1.007–1.085	TRUE	1.75E-11	1.725	1	0.1891
Family.FamilyXI.id.1936	Heel	IVW	7	−1.49E-02	5.84E-03	0.01	0.985	0.974–0.996	TRUE	1.12E−35	3.932	6	0.6859
Family.unknownfamily.id.1000006161	Heel	IVW	10	1.62E-02	6.11E-03	0.008	1.016	1.004–1.028	TRUE	1.02E-50	7.102	9	0.6265
Genus.*Eubacterium coprostanoligenes* group.id.11375	Heel	IVW	7	6.41E-02	1.76E-02	0.0002	1.066	1.029–1.103	TRUE	8.98E-31	10.72	6	0.0974
Genus.*Eisenbergiella*.id.11304	Heel	IVW	5	−3.67E-02	9.48E-03	0.0001	0.963	0.946–0.982	TRUE	2.13E-23	2.603	4	0.6263
Genus.*Rikenellaceae*RC9gutgroup.id.11191	Heel	IVW	6	−0.0113267	0.0084897	0.182	0.988	0.972–1.005	TRUE	5.98E−30	8.417	5	0.1347
Genus.RuminococcaceaeNK4A214group.id.11358	Heel	IVW	7	−3.16E-02	1.24E-02	0.011	0.968	0.945–0.992	FALSE	6.82E-01	2.221	6	0.8983
Genus.*Turicibacter*.id.2162.summary	Heel	IVW	3	−8.88E-02	1.51E-02	4.5 × 10^−9^	0.915	0.888–0.942	FALSE	4.17E-01	1.032	2	0.5969
Genus.unknowngenus.id.1000006162	Heel	IVW	9	1.86E-02	6.42E-03	0.003	1.018	1.005–1.031	TRUE	8.19E-46	5.682	8	0.6828
Order.Actinomycetales.id.420	Heel	IVW	2	4.50E-02	1.89E-02	0.017	1.046	1.007–1.085	TRUE	1.92E-11	1.714	1	0.1905
Order.NB1n.id.3953	Heel	IVW	10	1.62E-02	6.11E-03	0.008	1.016	1.004–1.028	TRUE	1.02E-50	7.102	9	0.6265

### Sensitivity test

After phasing out individual SNPs in the vast majority of studies, we discovered that the results did not change significantly, indicating the stability of our results to some extent. However, in studies where HE-BMD was used as an exposure, we discovered SNPs that significantly changed the results (outcome: genus Rikenellaceae RC9 gut group、 genus unknown-genus id 1,000,006,162, and genus *Turicibacter*) (Supplementary Figures S1–S3). We repeated the MR analysis step after eliminating the single SNPs that caused significant changes in the results (Supplementary Figures S4–S6). In the heterogeneity test, all *p*-values were greater than 0.05, indicating that the effect of heterogeneity could be ignored in our results. No evidence of horizontal pleiotropy in IVs was detected by the Pleiotrophy test (*p* > 0.05).

### MR Steiger directionality test

The results of the MR Steiger directionality test indicated two groups of studies with directional errors, which we excluded from the study (exposure: HE-BMD; outcome: genus *Turicibacter* and genus Ruminococcaceae NK4A214 group) (Table 5).

### Bonferroni correction

After Bonferroni correction with the threshold set at *p* < 5 × 10^−6^, the gut microbiota causally associated with LS-BMD was genus *Prevotella*; the gut microbiota causally associated with FN-BMD were phylum Lentisphaerae, family Prevotellaceae, and genus *Ruminococcus gauvreauii* group; the gut microbiota causally associated with FA-BMD was family Rikenellaceae; and the causally associated HE-BMD. The gut microbiota with a causal association with HE-BMD are order NB1n, genus *Eubacterium coprostanoligenes* group, and genus *Eisenbergiella*.

## Discussion

Numerous studies have shown that changes in the composition of gut microbiota play an important role in bone mineral density changes and the development of osteoporosis in humans. However, this is the first comprehensive study to look into whether there is a causal MR relationship between gut microbiota and bone density at four different skeletal sites. We used the largest summary statistics from GWAS for gut microbiota to determine causal relationships with BMD at four skeletal sites in over 400,000 Europeans from GEFOS and UKB. Our findings show that several gut microbiota genetic liabilities are causally associated with BMD at various skeletal sites, with multiple gut microbiota identified as protective factors or risk factors for BMD. The goal of this study is to use gut microbiota to gain new insights into the prevention and treatment of osteoporosis.

At a threshold level of *p* < 5 × 10^-6, our study identified six gut microbiota as protective factors and five as risk factors for FN-BMD. In the case of FA-BMD, 11 and 4 gut microbiota were classified as protective and risk factors, respectively. Regarding LS-BMD, six and eight gut microbiota were identified as protective and risk factors, respectively. For HE-BMD, two microbiota were protective, while six were risk factors. At a more stringent threshold of *p* < 5 × 10^-8, one gut microbiota was associated as a risk factor for FN-BMD, two for FA-BMD, four for LS-BMD, and three for HE-BMD, while four were protective for HE-BMD.

In observational studies, it is possible to determine to some extent whether gut microbiota is a protective or risk factor for BMD by comparing the relative abundance of gut microbiota in patients with that of the normal group, and the abundance of gut microbiota can be affected by a variety of factors, and the abundance of gut microbiota can provide a certain reference for the association between gut microbiota and BMD. Actinobacteria and Bifidobacteriacea were discovered to be BMD protective factors in our study, and Li, C et al. demonstrated that the abundance of Actinobacteria, which was more abundant in the normal-BMD group (Palacios-González et al., 2020), and Bifidobacteriaceae was positively correlated with BMD (Li et al., 2019). One study found that the genus *Escherichia Shigella* was more abundant in osteoporotic patients than in non-osteoporotic patients (Das et al., 2019); our study found a causal link between the genus *Escherichia shigella* and BMD.

Our study established a causal relationship between the Rikenellaceae family and its beneficial effects on bone mineral density (BMD); yet, previous research indicates a higher abundance of Rikenellaceae in cases of low BMD (Ozaki et al., 2021). Das et al. found Actinomyces to be more prevalent in individuals with osteoporosis compared with those with normal BMD (Das et al., 2019); a finding corroborated by our research, which also identifies Actinomyces as a BMD risk factor. Additionally, our results are consistent with previous studies indicating a greater prevalence of the Coriobacteriales order in osteoporotic patients compared with controls (He et al., 2020), suggesting its role as a BMD risk factor. In summary, while comparing previous observational studies supports our conclusions, discrepancies may arise from variations in population demographics, experimental methodologies, and confounding factors.

Our study highlights the significant causal association of the *Ruminococcus torques* group with various BMD sites, identifying it as a risk factor for LS-BMD, FA-BMD, and HE-BMD. Initially identified in 1974 (Holdeman and Moore, 1974), this bacterium, a member of the *Ruminococcus* genus (Holdeman and Moore, 1974), has been understudied in the context of BMD. Our findings indicate that *Ruminococcus* is a risk factor for BMD. Conversely, in our study, the *Ruminococcus gauvreauii* group and the *Ruminococcus gnavus* group, both from the same genus, emerged as beneficial for BMD. Additionally, Tyzzerella was causally linked to BMD risk factors at FN-BMD, LS-BMD, and FA-BMD. This genus, known for causing infectious diarrhea in mice due to Tyzzer’s disease since 1917 (Yutin and Galperin, 2013), has not been extensively studied for its impact on BMD.

Our findings show that the results of the association between BMD and gut microbiota differ in different skeletal sites. A previous study found that gut microbiota composition had a higher correlation with BMD values/T scores in the hip than in the lumbar spine (Huang et al., 2023). Only pelvic BMD was eventually found to have modest associations with gut microbiota in another Polygenic Risk Score (PRS) analysis, which was performed using 31 different sites of BMD as the outcome (Cheng et al., 2020). The studies discussed above provide evidence that the relationship between BMD-related microbiota and different skeletal sites may be inconsistent. However, research on the relationship of BMD with gut microbiota at various sites is still limited (Cheng et al., 2020). More research studies on the reasons for the differences in the association of BMD with gut microbiota at different sites is required in the future.

The mechanism by which the gut microbiota regulates changes in human bone density is still unknown. There are currently several points of view on the potential mechanism of gut microbiota for osteoporosis: intestinal barrier and intestinal flora dysbiosis: Intestinal flora dysbiosis can cause osteoporosis by affecting the intestinal barrier and impairing its function (Wang et al., 2022). Gut flora transplantation, on the other hand, improves intestinal barrier function and gut flora composition in osteoporosis, reducing bone loss (Ma et al., 2021). Short-chain fatty acids are metabolites produced by intestinal flora fermentation of resistant starches, dietary fiber, and other indigestible carbohydrates, and gut microbiota can play an important role in osteoclasts and osteoblasts by influencing short-chain fatty acids. (Corrêa-Oliveira et al., 2016; Wallimann et al., 2021; He and Chen, 2022). Another gut microbiota-derived metabolite trimethylamine N-oxide, a choline metabolite, has also been shown to promote adipogenic differentiation of bone marrow and mesenchymal stem cells (BMSCs) through upregulating NF-κB signaling pathway, inhibiting osteogenic differentiation, and resulting in bone loss and even osteoporosis (Hartwig et al., 2017; Lin et al., 2020).

Trabecular bone BMD distributions in adults are independent of skeletal site (Roschger et al., 2003, 2008), whereas mean mineral content of cortical bone varies by skeletal site (Roschger et al., 2008). Lumbar trabecular and cortical bone loss with age were responsible for 29.5 and 70.5% of total bone BMD (Sandor et al., 1992), respectively. Trabecular bone loss accounts for only approximately one-third of the total bone loss each year, implying that cortical bone loss is more significant in the context of increased osteoporosis risk (Sandor et al., 1992). It is unclear whether gut flora has a greater impact on cortical bone. However, given the importance of cortical bone loss in the progression of osteoporosis, correlations may be possible in the future.

Furthermore, one study found that men who engaged in weight-bearing exercise had greater bone density in the lumbar spine, face, and femoral neck, as well as a larger cortical cross-sectional area and trabecular bone (Nilsson et al., 2013). Therefore, a study on the association between gut microbiota and BMD could be carried out at a later date specifically for people who regularly perform weight-bearing exercise or other exercise, to explore whether exercise affects BMD by altering gut microbiota and thereby influencing BMD (Marshall et al., 2008). However, our study only included people of European descent, comparing differences in gut flora, and affecting cortical and trabecular bone between races could be a new avenue for future research.

Dual X-ray absorptiometry (DXA) confirms that peak bone mass (PBM) varies by site, for example, the hip tends to reach PBM before the spine (Berger et al., 2010). Gender plays a role in bone density variability at skeletal sites, as peak bone mass differs between genders. For example, in Canadian women aged 50 years and older, the prevalence of osteoporosis (T score < 2.5) is 12.0% (L1-L4) and 9% (Berger et al., 2010). In Canada, women aged 50 years and older have a prevalence of osteoporosis (T-score < −2.5) at 12.0% (L1-L4) and 9.1% (total hip), while men have a prevalence of 2.9% (L1-L4) and 0.9% (total hip) (Berger et al., 2010). The reason for the difference in BMD between genders may be due to a variety of factors, including ovarian secretion in women. Furthermore, we believe that gender differences in intestinal flora may be a contributing factor to BMD differences. Furthermore, aging is known to cause changes in gut flora, and bone loss increases with age. Moreover, does the gut flora that changes with age play an important role in direct bone loss? Or what role does gut flora play in regulating BMD in response to time factors? This requires additional research in the future.

The main causes of accelerated bone loss in women are increasing age and decreased ovarian secretion after menopause, which can lead to osteoporosis (Compston et al., 2019) and affect osteoclasts, osteoblasts, and osteocytes (Khosla et al., 2012; Manolagas et al., 2013). In patients with postmenopausal osteoporosis, there appears to be an association between estrogen levels and gut microbes, with the underlying mechanisms being a lack of estrogen, increased intestinal permeability, entry of bacterial products into the circulation (Hass et al., 2009), and the release of the pro-inflammatory cytokines TNF-a and IFN-g, which leads to further intestinal permeability (Patrick et al., 2006). Facilitates the body’s circulation of intestinal metabolic products and structural elements like endotoxins, short-chain fatty acids, and microbe-associated molecular patterns (Hernandez et al., 2016). Intestinal flora migrates to the lamina propria, promoting inflammatory processes, activating pro-inflammatory T-cells in the bone marrow, and influencing bone remodeling (Gomez et al., 2015; Yu et al., 2020).

According to studies, Actinobacteria and Lachnospira are less abundant in menopausal women than in pre-menopausal women, while Prevotella is more abundant (Santos-Marcos et al., 2018). Interestingly, these flora (or flora related to them) were found to be causally associated with BMD in our study. Female menopause is well known to be associated with a significant decrease in estrogen, and estrogen levels have been shown to regulate gut flora (Chen and Madak-Erdogan, 2016; Baker et al., 2017; Vieira et al., 2017). As a result, it is reasonable to assume that changes in the gut flora caused by altered estrogen levels, as well as the causal relationship between these altered gut flora and BMD, could eventually influence BMD, but we cannot rule out the influence of other factors during menopause.

Gut flora can influence human metabolomics studies via metabolic pathways, potentially affecting osteoporosis. Additionally, it has been demonstrated that altered amino acid metabolism may be the mediating factor in the correlation between gut microbes and osteoporosis, and that valine, leucine, and isoleucine degradation, as well as the metabolism of tyrosine and tryptophan, are significantly linked to both osteoporosis and microbiota biomarkers (Ling et al., 2021). Lumbar spine osteoporosis was linked to increased activity in bacterial pathways involving peptidases (Ling et al., 2021). The underlying mechanism is that bacteria use peptidases to break down proteins in the gut, producing tyrosine (Diether and Willing, 2019). Tyrosine metabolism can produce succinate, which can stimulate osteoclasts by binding to specific receptors on osteoclast cells, potentially increasing bone loss (Guo et al., 2017).

Tryptophan metabolism is linked to femoral neck osteoporosis (Ling et al., 2021), and the mechanism is thought to be related to factors such as gene transcription. Leucine intake is strongly linked to increased bone density in the spine and forearms (Jennings et al., 2016), and plasma leucine levels are strongly correlated with gut microbiota (Wu et al., 2020). The metabolic doctrine may account for the variability in causal associations between different bone density sites and gut microbiota (exposure) in our findings.

Research has demonstrated a negative correlation between the tryptophan and phenylalanine metabolic pathways and Lachnospiraceae (Zhang et al., 2021). It was also established in our study that there was a causal correlation between BMD and the flora Lachnospiraceae, and that this flora was one of the risk factors for BMD in the lumbar spine. Furthermore, there was a negative correlation between L-tryptophan and Tyzzerella (Zhang et al., 2021). Our findings not only demonstrated a causal relationship between Tyzzerella and BMD in the lumbar spine, forearm, and femoral neck but also indicated that this flora was one of the risk factors for BMD, which is entirely consistent with its negatively correlated phenylalanine metabolic profile. In conclusion, additional research is required to fully understand how the gut microbiota may contribute to the development of BMD linked to amino acid metabolism.

Our findings, however, have some limitations. The IVW method dominated our analysis of multiple IVs, and we did not use other MR methods to validate our results, which may be susceptible to non-robustness. Second, in this study, all of the subjects were of European origin, so extrapolating our findings to other ethnicities may not be appropriate, and more research with a diverse range of ethnicities is needed.

## Conclusion

In this study, we investigated the causal relationships between gut microbiota and BMD at four skeletal sites. Our findings revealed distinct causal directions: FN-BMD exhibited six positive and seven negative causal directions; FA-BMD showed seven positive and six negative causal directions; LS-BMD had six positive and 13 negative causal directions; and HE-BMD demonstrated six positive and nine negative causal directions. These results challenge the conclusions of some previous observational studies. Future research may require the analysis of more extensive Genome-Wide Association Studies (GWAS) data or advanced methodologies to corroborate our findings. Crucially, our research contributes new insights into the prevention and treatment of osteoporosis and osteopenia.

## Data availability statement

The original contributions presented in the study are included in the article/Supplementary material, further inquiries can be directed to the corresponding authors.

## Ethics statement

The manuscript presents research on animals that do not require ethical approval for their study.

## Author contributions

YX: Conceptualization, Data curation, Formal analysis, Investigation, Methodology, Resources, Software, Supervision, Writing – original draft, Writing – review & editing. XW: Supervision, Writing – review & editing. HL: Supervision, Writing – review & editing. JK: Supervision, Writing – review & editing. XL: Supervision, Writing – review & editing. AY: Writing – review & editing. ZD: Investigation, Writing – review & editing.

## Footnotes

^1^ https://mibiogen.gcc.rug.nl/menu/main/home/

^2^GEFOS, http://www.gefos.org/

^3^ https://gwas.mrcieu.ac.uk/datasets/

^4^ https://www.phenoscanner.medschl.cam.ac.uk/

## References

[ref1] Armstrong R. A. (2014). When to use the Bonferroni correction. Ophthalmic Physiol. Opt. 34, 502–508. doi: 10.1111/opo.12131, PMID: 24697967

[ref2] Aziziyeh R. Amin M. Habib M. Garcia Perlaza J. Szafranski K. McTavish R. K.. (2019). The burden of osteoporosis in four Latin American countries: Brazil, Mexico, Colombia, and Argentina. J. Med. Econ. 22, 638–644. doi: 10.1080/13696998.2019.1590843, PMID: 30835577

[ref3] Baker J. M. Al-Nakkash L. Herbst-Kralovetz M. M. (2017). Estrogen-gut microbiome axis: Physiological and clinical implications. Maturitas 103, 45–53. doi: 10.1016/j.maturitas.2017.06.025, PMID: 28778332

[ref4] Berger C. Goltzman D. Langsetmo L. Joseph L. Jackson S. Kreiger N.. (2010). Peak bone mass from longitudinal data: implications for the prevalence, pathophysiology, and diagnosis of osteoporosis. J. Bone Miner. Res. 25, 1948–1957. doi: 10.1002/jbmr.95, PMID: 20499378 PMC5101070

[ref5] Bowden J. Davey Smith G. Burgess S. (2015). Mendelian randomization with invalid instruments: effect estimation and bias detection through Egger regression. Int. J. Epidemiol. 44, 512–525. doi: 10.1093/ije/dyv080 26050253 PMC4469799

[ref6] Bowden J. Davey Smith G. Haycock P. C. Burgess S. (2016). Consistent Estimation in Mendelian Randomization with Some Invalid Instruments Using a Weighted Median Estimator. Genet. Epidemiol. 40, 304–314. doi: 10.1002/gepi.21965, PMID: 27061298 PMC4849733

[ref7] Burgess S. Butterworth A. Thompson S. G. (2013). Mendelian randomization analysis with multiple genetic variants using summarized data. Genet. Epidemiol. 37, 658–665. doi: 10.1002/gepi.21758, PMID: 24114802 PMC4377079

[ref8] Burgess S. Small D. S. Thompson S. G. (2017). A review of instrumental variable estimators for Mendelian randomization. Stat. Methods Med. Res. 26, 2333–2355. doi: 10.1177/0962280215597579, PMID: 26282889 PMC5642006

[ref9] Burgess S. Thompson S. G. (2017). Interpreting findings from Mendelian randomization using the MR-Egger method. Eur. J. Epidemiol. 32, 377–389. doi: 10.1007/s10654-017-0255-x, PMID: 28527048 PMC5506233

[ref10] Carding S. Verbeke K. Vipond D. T. Corfe B. M. Owen L. J. (2015). Dysbiosis of the gut microbiota in disease. Microb. Ecol. Health Dis. 26:26191. doi: 10.3402/mehd.v26.26191 25651997 PMC4315779

[ref11] Chen K. L. Madak-Erdogan Z. (2016). Estrogen and microbiota crosstalk: should we pay attention? Trends Endocrinol. Metab. 27, 752–755. doi: 10.1016/j.tem.2016.08.001, PMID: 27553057

[ref12] Cheng S. Qi X. Ma M. Zhang L. Cheng B. Liang C.. (2020). Assessing the Relationship Between Gut Microbiota and Bone Mineral Density. Front. Genet. 11:6. doi: 10.3389/fgene.2020.00006, PMID: 32082367 PMC7005253

[ref13] Clynes M. A. Harvey N. C. Curtis E. M. Fuggle N. R. Dennison E. M. Cooper C. (2020). The epidemiology of osteoporosis. Br. Med. Bull. 133, 105–117. doi: 10.1093/bmb/ldaa005, PMID: 32282039 PMC7115830

[ref14] Compston J. E. McClung M. R. Leslie W. D. (2019). Osteoporosis. Lancet 393, 364–376. doi: 10.1016/S0140-6736(18)32112-3 30696576

[ref15] Corrêa-Oliveira R. Fachi J. L. Vieira A. Sato F. T. Vinolo M. A. (2016). Regulation of immune cell function by short-chain fatty acids. Clin. Transl. Immunol. 5:e73. doi: 10.1038/cti.2016.17, PMID: 27195116 PMC4855267

[ref16] Darbà J. Kaskens L. Pérez-Álvarez N. Palacios S. Neyro J. L. Rejas J. (2015). Disability-adjusted-life-years losses in postmenopausal women with osteoporosis: a burden of illness study. BMC Public Health 15:324. doi: 10.1186/s12889-015-1684-7, PMID: 25880810 PMC4392468

[ref17] Das M. Cronin O. Keohane D. M. Cormac E. M. Nugent H. Nugent M.. (2019). Gut microbiota alterations associated with reduced bone mineral density in older adults. Rheumatology (Oxford) 58, 2295–2304. doi: 10.1093/rheumatology/kez302, PMID: 31378815 PMC6880854

[ref18] Davey Smith G. Hemani G. (2014). Mendelian randomization: genetic anchors for causal inference in epidemiological studies. Hum. Mol. Genet. 23, R89–R98. doi: 10.1093/hmg/ddu328 25064373 PMC4170722

[ref19] Diether N. E. Willing B. P. (2019). Microbial Fermentation of Dietary Protein: An Important Factor in Diet^−^Microbe^−^Host Interaction. Microorganisms 7:19. doi: 10.3390/microorganisms7010019, PMID: 30642098 PMC6352118

[ref20] Fischer S. Kapinos K. A. Mulcahy A. Pinto L. Hayden O. Barron R. (2017). Estimating the long-term functional burden of osteoporosis-related fractures. Osteoporos. Int. 28, 2843–2851. doi: 10.1007/s00198-017-4110-4, PMID: 28647804

[ref21] Gomez A. Luckey D. Taneja V. (2015). The gut microbiome in autoimmunity: Sex matters. Clin. Immunol. 159, 154–162. doi: 10.1016/j.clim.2015.04.016, PMID: 25956531 PMC4560596

[ref22] Guo Y. Xie C. Li X. Yang J. Yu T. Zhang R.. (2017). Succinate and its G-protein-coupled receptor stimulates osteoclastogenesis. Nat. Commun. 8:15621. doi: 10.1038/ncomms15621, PMID: 28561074 PMC5460032

[ref23] Hartwig F. P. Davey Smith G. Bowden J. (2017). Robust inference in summary data Mendelian randomization via the zero modal pleiotropy assumption. Int. J. Epidemiol. 46, 1985–1998. doi: 10.1093/ije/dyx102, PMID: 29040600 PMC5837715

[ref24] Hass M. A. Nichol P. Lee L. Levin R. M. (2009). Estrogen modulates permeability and prostaglandin levels in the rabbit urinary bladder. Prostaglandins Leukot. Essent. Fatty Acids 80, 125–129. doi: 10.1016/j.plefa.2008.11.010, PMID: 19181506

[ref25] He Y. X. Chen Y. X. (2022). The potential mechanism of the microbiota-gut-bone axis in osteoporosis: a review. Osteoporos. Int. 33, 2495–2506. doi: 10.1007/s00198-022-06557-x, PMID: 36169678

[ref26] He J. Q. Xu S. Zhang B. Xiao C. Chen Z. Si F.. (2020). Gut microbiota and metabolite alterations associated with reduced bone mineral density or bone metabolic indexes in postmenopausal osteoporosis. Aging (Albany NY) 12, 8583–8604. doi: 10.18632/aging.103168, PMID: 32392181 PMC7244073

[ref27] Hernandez C. J. Guss J. D. Luna M. Goldring S. R. (2016). Links Between the Microbiome and Bone. J. Bone Miner. Res. 31, 1638–1646. doi: 10.1002/jbmr.2887, PMID: 27317164 PMC5434873

[ref28] Holdeman L. V. Moore W. E. C. (1974). New Genus, Coprococcus, Twelve New Species, and Emended Descriptions of Four Previously Described Species of Bacteria from Human Feces. Int. J. Syst. Evol. Microbiol. 24, 260–277. doi: 10.1099/00207713-24-2-260

[ref29] Holmes M. V. Ala-Korpela M. Smith G. D. (2017). Mendelian randomization in cardiometabolic disease: challenges in evaluating causality. Nat. Rev. Cardiol. 14, 577–590. doi: 10.1038/nrcardio.2017.78, PMID: 28569269 PMC5600813

[ref30] Huang D. Wang J. Zeng Y. Li Q. Wang Y. (2023). Identifying microbial signatures for patients with postmenopausal osteoporosis using gut microbiota analyses and feature selection approaches. Front. Microbiol. 14:1113174. doi: 10.3389/fmicb.2023.1113174 37077242 PMC10106639

[ref31] Jennings A. MacGregor A. Spector T. Cassidy A. (2016). Amino acid intakes are associated with bone mineral density and prevalence of low bone mass in women: evidence from discordant monozygotic twins. J. Bone Miner. Res. 31, 326–335. doi: 10.1002/jbmr.2703, PMID: 26334651 PMC4832262

[ref32] Keller-Baruch J. Forgetta V. Manousaki D. Zhou S. R. Richards J. B. (2020). Genetically Decreased Circulating Vascular Endothelial Growth Factor and Osteoporosis Outcomes: A Mendelian Randomization Study. J. Bone Miner. Res. 35, 649–656. doi: 10.1002/jbmr.3937, PMID: 31821593

[ref33] Khosla S. Oursler M. J. Monroe D. G. (2012). Estrogen and the skeleton. Trends Endocrinol. Metab. 23, 576–581. doi: 10.1016/j.tem.2012.03.008, PMID: 22595550 PMC3424385

[ref34] Kurilshikov A. Medina-Gomez C. Bacigalupe R. Radjabzadeh D. Wang J. Demirkan A.. (2021). Large-scale association analyses identify host factors influencing human gut microbiome composition. Nat. Genet. 53, 156–165. doi: 10.1038/s41588-020-00763-1, PMID: 33462485 PMC8515199

[ref35] Li C. Huang Q. Yang R. Dai Y. Zeng Y. Tao L.. (2019). Gut microbiota composition and bone mineral loss-epidemiologic evidence from individuals in Wuhan. China. Osteoporos Int 30, 1003–1013. doi: 10.1007/s00198-019-04855-5, PMID: 30666372

[ref36] Lin H. Liu T. Li X. Gao X. Wu T. Li P. (2020). The role of gut microbiota metabolite trimethylamine N-oxide in functional impairment of bone marrow mesenchymal stem cells in osteoporosis disease. Ann. Transl. Med. 8:1009. doi: 10.21037/atm-20-5307, PMID: 32953809 PMC7475507

[ref37] Ling C. W. Miao Z. Xiao M. L. Zhou H. Jiang Z. Fu Y.. (2021). The association of gut microbiota with osteoporosis is mediated by amino acid metabolism: multiomics in a large cohort. J. Clin. Endocrinol. Metab. 106, e3852–e3864. doi: 10.1210/clinem/dgab492 34214160

[ref38] Ma S. Wang N. Zhang P. Wu W. Fu L. (2021). Fecal microbiota transplantation mitigates bone loss by improving gut microbiome composition and gut barrier function in aged rats. PeerJ 9:e12293. doi: 10.7717/peerj.12293, PMID: 34721980 PMC8542369

[ref39] Manolagas S. C. O'Brien C. A. Almeida M. (2013). The role of estrogen and androgen receptors in bone health and disease. Nat. Rev. Endocrinol. 9, 699–712. doi: 10.1038/nrendo.2013.179, PMID: 24042328 PMC3971652

[ref40] Marshall L. M. Zmuda J. M. Chan B. K. S. Barrett-Connor E. Cauley J. A. Ensrud K. E.. (2008). Race and ethnic variation in proximal femur structure and BMD among older men. J. Bone Miner. Res. 23, 121–130. doi: 10.1359/jbmr.070908, PMID: 17892375 PMC2663587

[ref41] Martinez-Gil N. Patino-Salazar J. D. Rabionet R. Grinberg D. Balcells S. (2023). Genome-wide association studies (GWAS) vs functional validation: the challenge of the post-GWAS era. Rev. Osteoporos. 15, 29–39,

[ref42] Morris J. A. Kemp J. P. Youlten S. E. Laurent L. Logan J. G. Chai R. C.. (2019). Author Correction: An atlas of genetic influences on osteoporosis in humans and mice. Nat. Genet. 51:920. doi: 10.1038/s41588-019-0415-x, PMID: 30988516

[ref43] Nicholson J. K. Holmes E. Kinross J. Burcelin R. Gibson G. Jia W.. (2012). Host-gut microbiota metabolic interactions. Science 336, 1262–1267. doi: 10.1126/science.1223813 22674330

[ref44] NIH Consensus Development Panel on Osteoporosis Prevention, Diagnosis, and Therapy (2001). Osteoporosis prevention, diagnosis, and therapy. JAMA 285, 785–795. doi: 10.1001/jama.285.6.785 11176917

[ref45] Nilsson M. Ohlsson C. Mellström D. Lorentzon M. (2013). Sport-specific association between exercise loading and the density, geometry, and microstructure of weight-bearing bone in young adult men. Osteoporos. Int. 24, 1613–1622. doi: 10.1007/s00198-012-2142-3, PMID: 23011682 PMC3627855

[ref46] Notelovitz M. (1993). Osteoporosis-screening, prevention, and management. Fertil. Steril. 59, 707–725. doi: 10.1016/S0015-0282(16)55848-8 8458485

[ref47] Ozaki D. Kubota R. Maeno T. Abdelhakim M. Hitosugi N. (2021). Association between gut microbiota, bone metabolism, and fracture risk in postmenopausal Japanese women. Osteoporos. Int. 32, 145–156. doi: 10.1007/s00198-020-05728-y, PMID: 33241467 PMC7755620

[ref48] Palacios-González B. Ramírez-Salazar E. G. Rivera-Paredez B. Quiterio M. Flores Y. N. Macias-Kauffer L.. (2020). A Multi-Omic Analysis for Low Bone Mineral Density in Postmenopausal Women Suggests a RELATIONSHIP between Diet, Metabolites, and Microbiota. Microorganisms 8:630. doi: 10.3390/microorganisms8111630, PMID: 33105628 PMC7690388

[ref49] Patrick D. M. Leone A. K. Shellenberger J. J. Dudowicz K. A. King J. M. (2006). Proinflammatory cytokines tumor necrosis factor-alpha and interferon-gamma modulate epithelial barrier function in Madin-Darby canine kidney cells through mitogen activated protein kinase signaling. BMC Physiol. 6:2. doi: 10.1186/1472-6793-6-2, PMID: 16504032 PMC1402323

[ref50] Rettedal E. A. Ilesanmi-Oyelere B. L. Roy N. C. Coad J. Kruger M. C. (2021). The Gut Microbiome Is Altered in Postmenopausal Women With Osteoporosis and Osteopenia. JBMR Plus 5:e10452. doi: 10.1002/jbm4.10452, PMID: 33778322 PMC7990138

[ref51] Rinninella E. Raoul P. Cintoni M. Franceschi F. Miggiano G. Gasbarrini A.. (2019). What is the Healthy Gut Microbiota Composition? A Changing Ecosystem across Age, Environment, Diet, and Diseases. Microorganisms 7:10014. doi: 10.3390/microorganisms7010014, PMID: 30634578 PMC6351938

[ref52] Roschger P. Gupta H. S. Berzlanovich A. Ittner G. Dempster D. W. Fratzl P.. (2003). Constant mineralization density distribution in cancellous human bone. Bone 32, 316–323. doi: 10.1016/S8756-3282(02)00973-0, PMID: 12667560

[ref53] Roschger P. Paschalis E. P. Fratzl P. Klaushofer K. (2008). Bone mineralization density distribution in health and disease. Bone 42, 456–466. doi: 10.1016/j.bone.2007.10.021, PMID: 18096457

[ref54] Rothman K. J. (1990). No adjustments are needed for multiple comparisons. Epidemiology 1, 43–46. doi: 10.1097/00001648-199001000-00010 2081237

[ref55] Sandor T. Felsenberg D. Kalender W. A. Clain A. Brown E. (1992). Compact and trabecular components of the spine using quantitative computed tomography. Calcif. Tissue Int. 50, 502–506. doi: 10.1007/BF00582162, PMID: 1525704

[ref56] Santos-Marcos J. A. Rangel-Zuñiga O. A. Jimenez-Lucena R. Quintana-Navarro G. M. Garcia-Carpintero S. Malagon M. M.. (2018). Influence of gender and menopausal status on gut microbiota. Maturitas 116, 43–53. doi: 10.1016/j.maturitas.2018.07.008 30244778

[ref57] Sekula P. Del Greco M. F. Pattaro C. Köttgen A. (2016). Mendelian Randomization as an Approach to Assess Causality Using Observational Data. J. Am. Soc. Nephrol. 27, 3253–3265. doi: 10.1681/ASN.2016010098, PMID: 27486138 PMC5084898

[ref58] Sjogren K. Engdahl C. Henning P. Lerner U. H. Tremaroli V. Lagerquist M. K.. (2012). The gut microbiota regulates bone mass in mice. J. Bone Miner. Res. 27, 1357–1367. doi: 10.1002/jbmr.1588, PMID: 22407806 PMC3415623

[ref59] Smith G. D. Ebrahim S. (2003). 'Mendelian randomization': can genetic epidemiology contribute to understanding environmental determinants of disease? Int. J. Epidemiol. 32, 1–22. doi: 10.1093/ije/dyg070, PMID: 12689998

[ref60] Smith G. D. Ebrahim S. (2004). Mendelian randomization: prospects, potentials, and limitations. Int. J. Epidemiol. 33, 30–42. doi: 10.1093/ije/dyh132, PMID: 15075143

[ref61] Staiger D. Stock J. H. (1997). Instrumental variables regression with weak instruments. Econometrica 65, 557–586. doi: 10.2307/2171753

[ref62] The Wellcome Trust Case Control Consortium (2007). Genome-wide association study of 14, 000 cases of seven common diseases and 3,000 shared controls. Nature 447, 661–678. doi: 10.1038/nature05911 17554300 PMC2719288

[ref63] Timpson N. J. Wade K. H. Smith G. D. (2012). Mendelian randomization: application to cardiovascular disease. Curr. Hypertens. Rep. 14, 29–37. doi: 10.1007/s11906-011-0242-7 22161218

[ref64] Verbanck M. Chen C. Y. Neale B. Do R. (2018). Detection of widespread horizontal pleiotropy in causal relationships inferred from Mendelian randomization between complex traits and diseases. Nat. Genet. 50, 693–698. doi: 10.1038/s41588-018-0099-7, PMID: 29686387 PMC6083837

[ref65] Vieira A. T. Castelo P. M. Ribeiro D. A. Ferreira C. M. (2017). Influence of oral and gut microbiota in the health of menopausal women. Front. Microbiol. 8:1884. doi: 10.3389/fmicb.2017.01884, PMID: 29033921 PMC5625026

[ref66] Visscher P. M. Wray N. R. Zhang Q. Sklar P. McCarthy M. I. Brown M. A.. (2017). 10 Years of GWAS Discovery: Biology, Function, and Translation. Am. J. Hum. Genet. 101, 5–22. doi: 10.1016/j.ajhg.2017.06.005, PMID: 28686856 PMC5501872

[ref67] Wallimann A. Magrath W. Thompson K. Moriarty T. Richards R. G. Akdis C. A.. (2021). Gut microbial-derived short-chain fatty acids and bone: a potential role in fracture healing. Eur. Cell. Mater. 41, 454–470. doi: 10.22203/eCM.v041a29, PMID: 33881768 PMC9100835

[ref68] Wang J. Kurilshikov A. Radjabzadeh D. Turpin W. Croitoru K. Bonder M. J.. (2018). Meta-analysis of human genome-microbiome association studies: the MiBioGen consortium initiative. Microbiome 6:101. doi: 10.1186/s40168-018-0479-3, PMID: 29880062 PMC5992867

[ref69] Wang N. Ma S. Fu L. (2022). Gut Microbiota Dysbiosis as One Cause of Osteoporosis by Impairing Intestinal Barrier Function. Calcif. Tissue Int. 110, 225–235. doi: 10.1007/s00223-021-00911-7, PMID: 34480200

[ref70] Wang J. H. Wang Y. Gao W. Wang B. Zhao H. Zeng Y.. (2017). Diversity analysis of gut microbiota in osteoporosis and osteopenia patients. Peerj 5:e3450. doi: 10.7717/peerj.3450, PMID: 28630804 PMC5474093

[ref71] Willers C. Norton N. Harvey N. C. Jacobson T. Johansson H. Lorentzon M.. (2022). Osteoporosis in Europe: a compendium of country-specific reports. Arch. Osteoporos. 17:23. doi: 10.1007/s11657-021-00969-8, PMID: 35079919 PMC8789736

[ref72] Wright N. C. Looker A. C. Saag K. G. Curtis J. R. Delzell E. S. Randall S.. (2014). The Recent Prevalence of Osteoporosis and Low Bone Mass in the United States Based on Bone Mineral Density at the Femoral Neck or Lumbar Spine. J. Bone Miner. Res. 29, 2520–2526. doi: 10.1002/jbmr.2269, PMID: 24771492 PMC4757905

[ref73] Wu W. Zhang L. Xia B. Tang S. Liu L. Xie J.. (2020). Bioregional alterations in gut microbiome contribute to the plasma metabolomic changes in pigs fed with inulin. Microorganisms 8:10111. doi: 10.3390/microorganisms8010111, PMID: 31941086 PMC7022628

[ref74] Yu M. Malik Tyagi A. Li J. Y. Adams J. Denning T. L. Weitzmann M. N.. (2020). PTH induces bone loss via microbial-dependent expansion of intestinal TNF(+) T cells and Th17 cells. Nat. Commun. 11:468. doi: 10.1038/s41467-019-14148-4, PMID: 31980603 PMC6981196

[ref75] Yutin N. Galperin M. Y. (2013). A genomic update on clostridial phylogeny: Gram-negative spore formers and other misplaced clostridia. Environ. Microbiol. 15, 2631–2641. doi: 10.1111/1462-2920.12173, PMID: 23834245 PMC4056668

[ref76] Zhang Q. Zhang Y. Zeng L. Chen G. Zhang L. Liu M.. (2021). The role of gut microbiota and microbiota-related serum metabolites in the progression of diabetic kidney disease. Front. Pharmacol. 12:757508. doi: 10.3389/fphar.2021.757508, PMID: 34899312 PMC8652004

[ref77] Zheng H. F. Forgetta V. Hsu Y. H. Estrada K. Rosello-Diez A. Leo P. J.. (2015). Whole-genome sequencing identifies EN1 as a determinant of bone density and fracture. Nature 526, 112–117. doi: 10.1038/nature14878, PMID: 26367794 PMC4755714

