# Supplementary material for: Assessment of the relationship between gut microbiota and bone mineral density: a two-sample Mendelian randomization study
Source: Front Microbiol. 2024 May 22;15:1298838. doi: 10.3389/fmicb.2024.1298838 (PMC11150656; doi:10.3389/fmicb.2024.1298838)

**Fig S1 SNPs that significantly changed the results （genus Rikenellaceae RC9 and HE-BMD）**


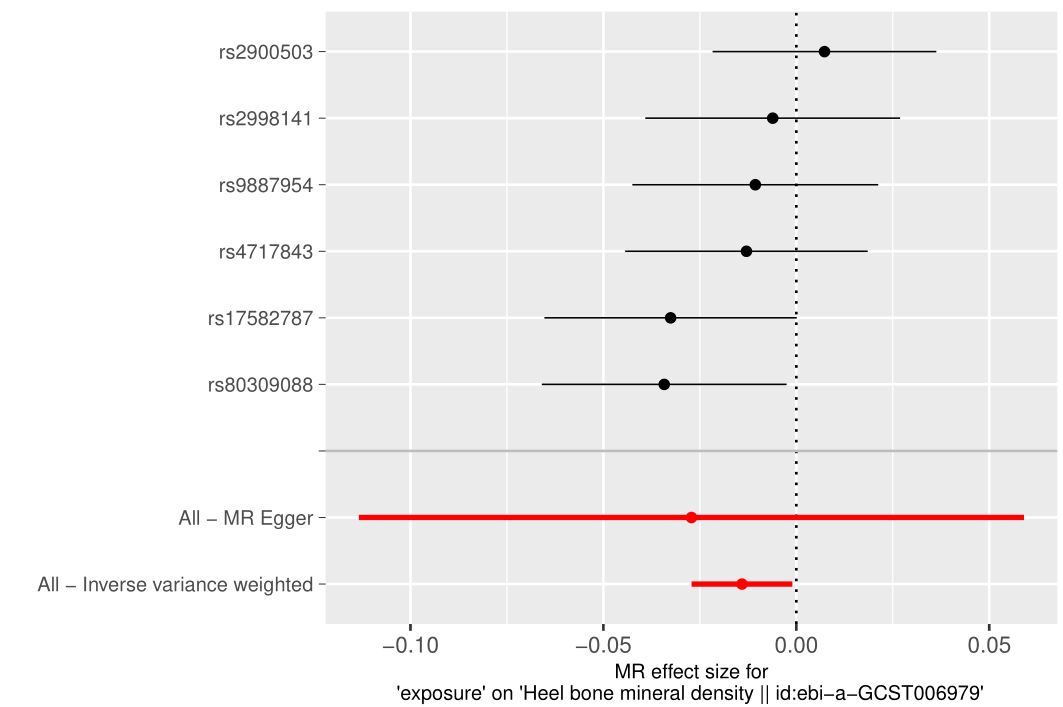


**Fig S2 SNPs that significantly changed the results （genus unknowngenus id 1000006162 and HE-BMD）**


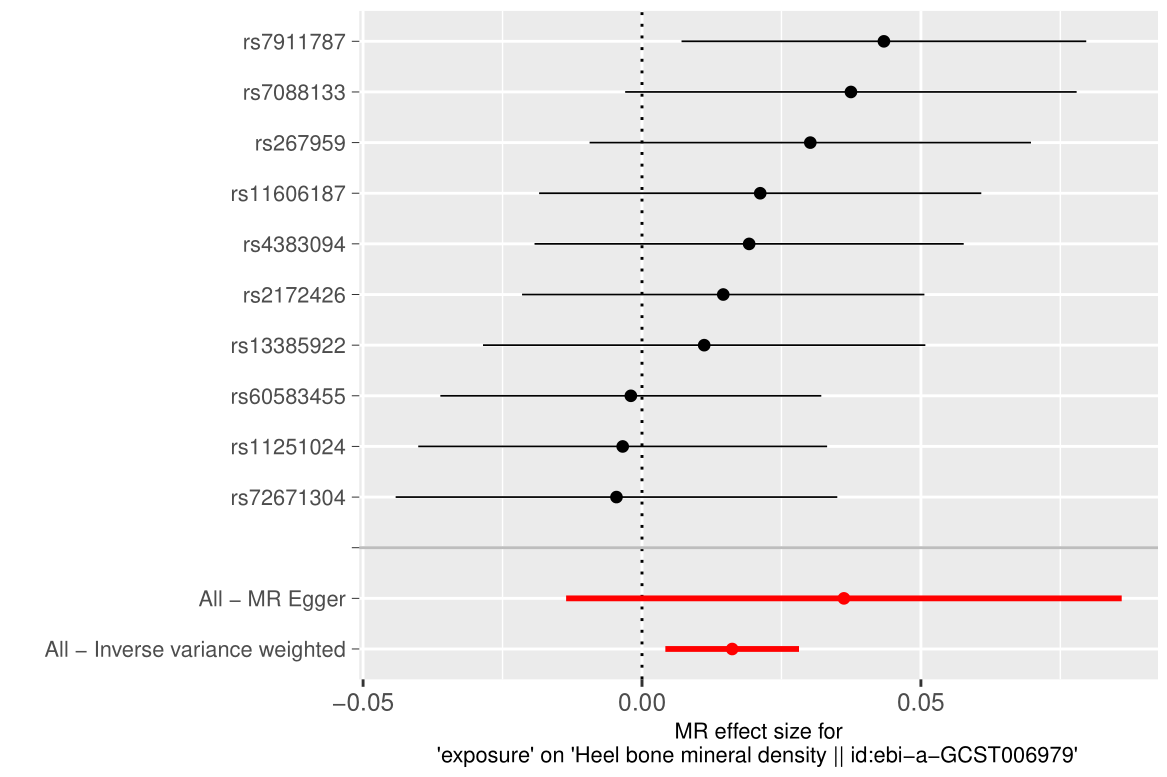


**Fig S3 SNPs that significantly changed the results （genus.Turicibacter and HE-BMD）**


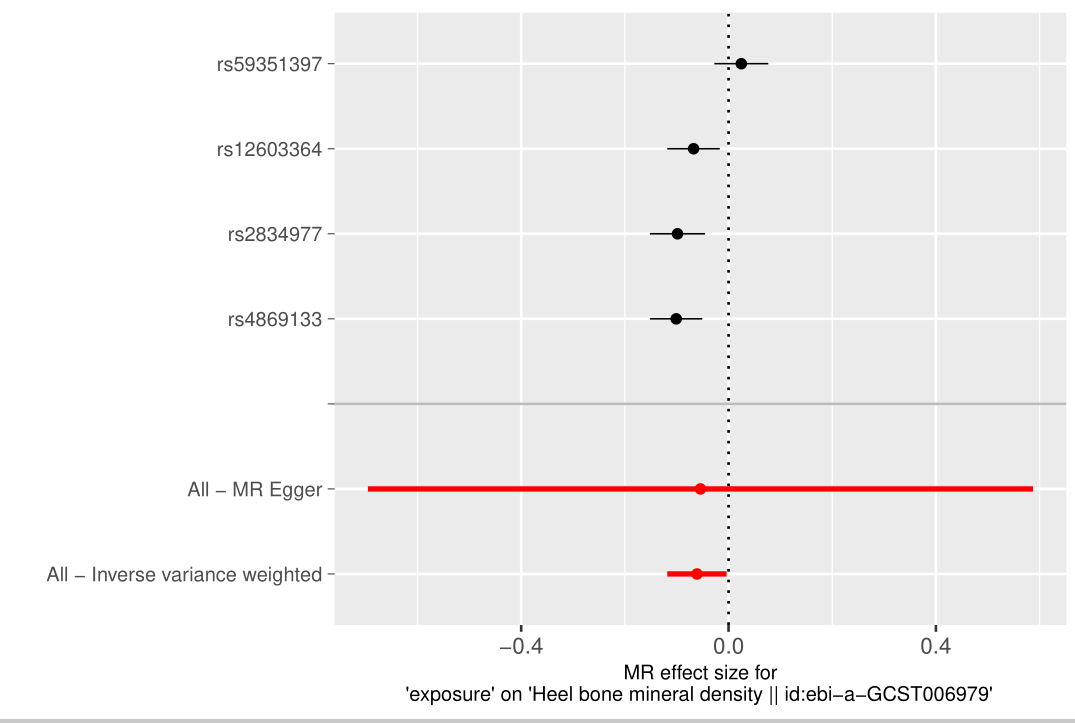


**Fig S4 Romved SNPs that significantly changed the results （genus Rikenellaceae RC9 and HE-BMD）**


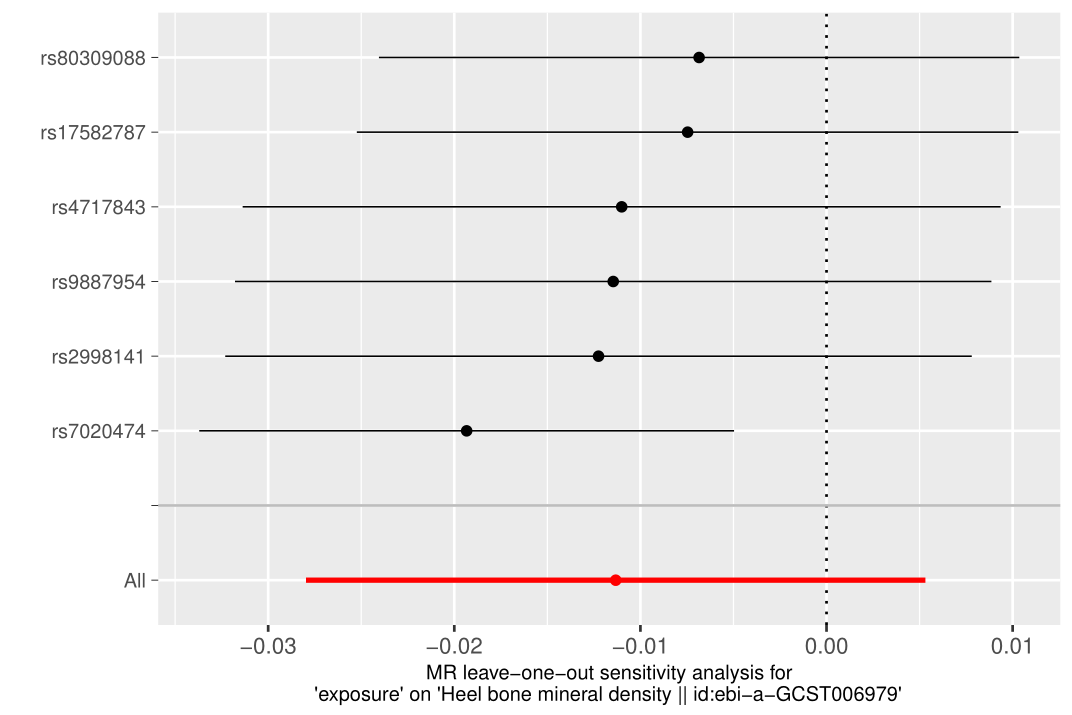


**Fig S5 Removed SNPs that significantly changed the results （genus unknowngenus id 1000006162 and HE-BMD）**


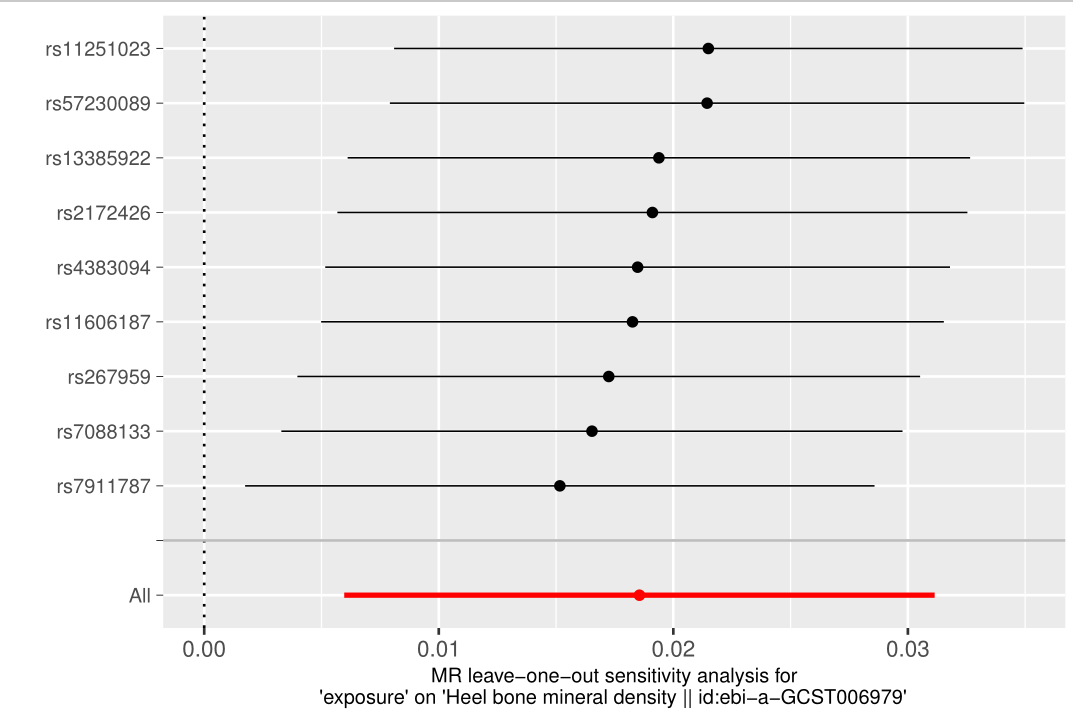


**Fig S6 Removed SNPs that significantly changed the results （genus.Turicibacter and HE-BMD）**


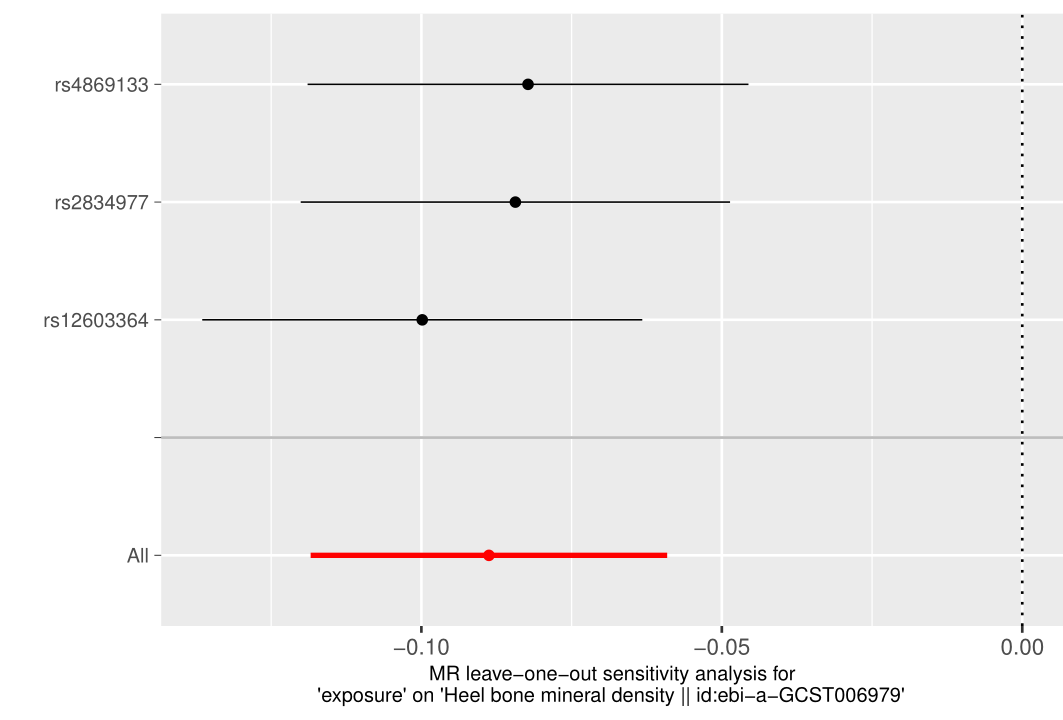

Supplement: Supplementary file 1 [file Data_Sheet_1.docx]
